# An Ecologist‐Friendly R Workflow for Expediting Species‐Level Classification of Camera Trap Images

**DOI:** 10.1002/ece3.70544

**Published:** 2024-12-12

**Authors:** L. Petroni, L. Natucci, A. Massolo

**Affiliations:** ^1^ Ethology Unit, Department of Biology University of Pisa Pisa Italy; ^2^ Faculty of Veterinary Medicine University of Calgary Calgary Alberta Canada; ^3^ UMR CNRS 6249 Chrono‐Environnement Université Bourgogne Franche‐Comté Besançon France

**Keywords:** camera trap, deep learning, image classification, R script, workflow, YOLO

## Abstract

Camera trapping has become increasingly common in ecological studies, but is hindered by analyzing large datasets. Recently, artificial intelligence (deep learning models in particular) has emerged as a promising solution. However, applying deep learning for images processing is complex and often requires programming skills in Python, reducing its accessibility. Some authors addressed this issue with user‐friendly software, and a further progress was the transposition of deep learning to R, a statistical language frequently used by ecologists, enhancing flexibility and customization of deep learning models without advanced computer expertise. We aimed to develop a user‐friendly workflow based on R scripts to streamline the entire process, from selecting to classifying camera trap images. Our workflow integrates the MegaDetector object detector for labelling images and custom training of the state‐of‐the‐art YOLOv8 model, together with potential for offline image augmentation to manage imbalanced datasets. Inference results are stored in a database compatible with Timelapse for quality checking of model predictions. We tested our workflow on images collected within a project targeting medium and large mammals of Central Italy, and obtained an overall precision of 0.962, a recall of 0.945, and a mean average precision of 0.913 for a training set of only 1000 pictures per species. Furthermore, the custom model achieved 91.8% of correct species‐level classifications on a set of unclassified images, reaching 97.1% for those classified with > 90% confidence. YOLO, a fast and light deep learning architecture, enables application of the workflow even on resource‐limited machines, and integration with image augmentation makes it useful even during early stages of data collection. All R scripts and pretrained models are available to enable adaptation of the workflow to other contexts, plus further development.

## Graphical abstract

Our manuscript presents a R workflow to streamline camera trap images classification at the species level, combining image augmentation and deep learning models in a user‐friendly environment. Tested on a dataset from Central Italy, the procedure achieved over 90% accuracy in classifying 17 target mammals, humans, and vehicles, starting from just 1000 images per category, significantly reducing classification time compared to manual methods.

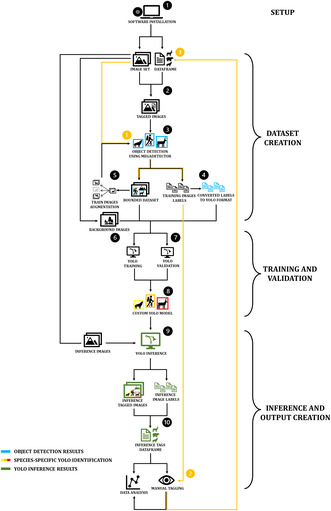

## 1 Introduction

The advent of camera trapping has profoundly impacted ecology studies, especially those on wildlife (Burton et al. 2015; Delisle et al. 2021). Camera trap versatility and cost‐effectiveness favored development of long term projects relying on many cameras continuously collecting data (Delisle et al. 2021). However, the increased image collection has also posed new challenges in terms of data analysis, with artificial intelligence emerging as a promising solution (Tuia et al. 2022).

Deep learning models seemed particularly suited for the task, and recent studies have demonstrated their power in reducing the manual work needed to classify camera trap images (Norouzzadeh et al. 2018; Tabak et al. 2019). Some pretrained models are publicly available for ecologists to download or in cloud‐based platforms such as Agouti (https://www.agouti.eu/). However, custom training has remained fundamental to obtain reliable results for distinct arrays of species, specific habitats, and camera positionings, given the limited ability of these pretrained models to generalize to new contexts (Beery, van Horn, and Perona 2018; Schneider et al. 2020).

Selecting the appropriate model for training can be challenging and should balance speed, accuracy, and ease of customization. One noteworthy architecture is YOLO (You Only Look Once), capable of processing images at high speeds while maintaining competitive accuracy, even with limited computational resources (Diwan, Anirudh, and Tembhurne 2023) making YOLO models appealing for studies where rapid tagging of large image datasets is crucial. Although initial studies implementing early versions have had reduced accuracy compared to other deep learning models (Schneider, Taylor, and Kremer 2018), more recent releases have performed comparably to other methods (Nguyen et al. 2023; Tan et al. 2022).

Another critical issue for such applications is the level of expertise required, as programming skills in Python have often been necessary (Tabak et al. 2020). This has been addressed by releasing user‐friendly software to perform deep learning tasks, in some cases even implementing YOLO‐based classifiers (Rigoudy et al. 2023), but they have generally lacked versatility for customization. Further progress has been the transposition of deep learning from Python to R (R Core Team 2023), such as MLWIC2 (Tabak et al. 2020), a package featuring functions for both inference and training, or the more flexible scripts implemented by Böhner et al. (2023). However, none of them have implemented YOLO.

Our objective was to provide ecologists with a simple protocol in R to train YOLO classifiers on custom camera trap datasets, without requiring advanced programming skills. Specifically, we aimed to: (i) define a workflow from establishment of working environments and image handling, to model training, validation, and inference on new data; and (ii) test the workflow by training a custom YOLO model to classify images of mammals obtained within a case study in Northern Apennines, Italy.

## 2 Materials and Methods

### 2.1 The Workflow

**FIGURE 1:**
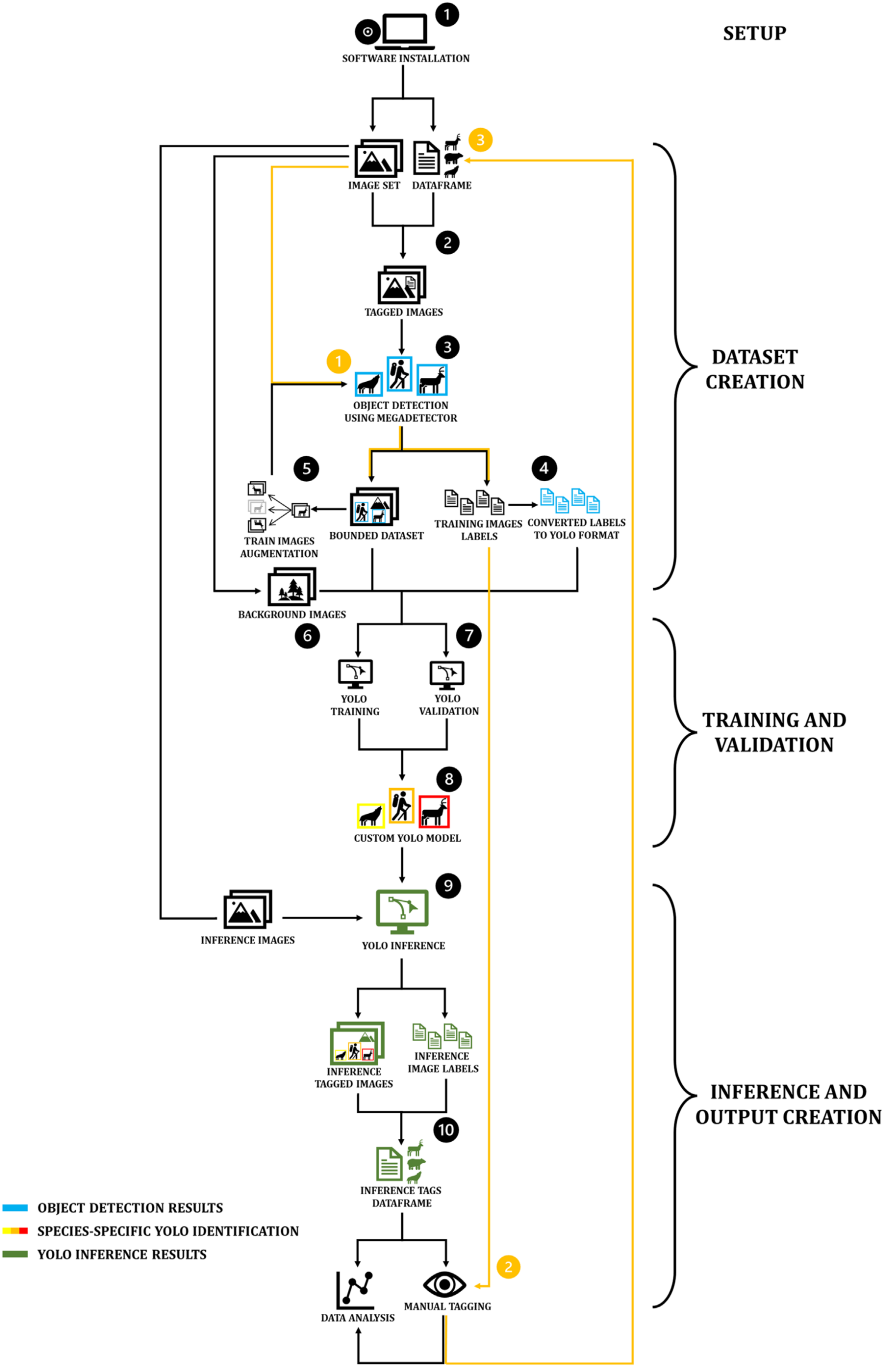
Ten‐stages workflow (in black) from establishment of working environments and image handling to model training, validation, and inference on new data. The flow for assisted tagging (in orange), including use of MegaDetector to detect objects in unprocessed images, followed by manual tagging.

The semi‐automated workflow unfolds in 10 sequential stages fully implemented in R (Figure 1); it interfaces with Python and Exiftool through the *reticulate* (v.1.31) and *exiftoolr* (v.0.2.3) packages, respectively (O'Brien 2023; Ushey, Allaire, and Tang 2024). We developed the code in Windows 11, following installation of Anaconda (https://www.anaconda.com), Git for Windows (https://git‐scm.com), and Exiftool (https://exiftool.org/).

The process integrated two deep learning models: (i) MegaDetector version 5.0a (Beery et al. 2019), an object detector based on the YOLOv5 architecture used to generate bounding boxes around subjects; and (ii) YOLOv8m (Jocher, Chaurasia, and Qiu 2023) as the starting point for custom training and inference. All stages were performed on a laptop (Intel Core i7‐12700H, NVIDIA GeForce RTX 3070 Laptop GPU, and 16 Gb RAM). Further details on hardware and software releases are provided in the workflow scripts (Data1).

Applying the workflow only requires users to provide proper locations of software, folders and files in the workflow script (Data 1—Workflow). Then, just two conditions are essential for custom training: (i) some images must have been previously classified; and (ii) species‐level classification should be stored in a .csv database that includes the full image path, picture type, and the timestamp of image capture (example database in Data 1).

### 2.2 Stages 1–2

The first stage defines the working environments and downloads all necessary packages to execute the workflow. It must be carried out the first time the workflow is executed, whereas it is not necessary for subsequent implementations. In Stage 2, the user defines the target species and establishes a fixed number representing the required total images per species, encompassing both training and validation. Then, based on species classification, a function copies and renames the original pictures into a user‐specified folder. Such a function is designed to pick images as balanced as possible among species and day‐night conditions. In detail, only single‐species pictures are included in training and validation sets to reduce the risk of misidentifications. Black/white and color pictures selected are balanced based on sunset and sunrise times, as this may improve model performance (Tan et al. 2022). When the requested number of images is not available, all pictures of a species are included. Gathering extensive data can be challenging, especially for species at low densities, and we allow to merge phenotypically similar species in higher‐order categories (e.g., *Martes martes* , *M. foina* and *Mustela putorius* ) to reduce the risk of missing the rarest species (Tabak et al. 2020). Furthermore, it is also possible to perform image augmentation (see Section 2.4).

### 2.3 Stages 3–4

In Stage 3, the MegaDetector model automatically generates bounding boxes around the subjects in the images. Boxes are not validated by hand, but the confidence level for accepting a classification was set to 0.50 to reduce false positives. In Stage 4, coordinates of the boxes are converted to a YOLO‐compatible format and stored in single files for each picture (labels), together with the id of the species derived from image names. Finally, images without detections are removed.

### 2.4 Stage 5

Imbalanced datasets are common due to differences in camera placement and specifications, and even habitat features at sampling sites (Hofmeester et al. 2019). As this may bias model performance, a common mitigation is to increase the size of the dataset by applying transformations to the existing data, a practice called data augmentation (Shorten and Khoshgoftaar 2019). Offline augmentation is performed through the *magick* package (v.2.8.1) to expand the size of the image set prior to model training (Ooms 2024; Shorten and Khoshgoftaar 2019). Specifically, offline augmentation is conducted subsequently to object detection to increase the probability of detecting objects even in the synthetic images. Moreover, it only targets the species with fewer pictures, doubling their count at each transformation until reaching the number indicated in Stage 2. Then, Stages 3 and 4 are repeated to generate bounding boxes and labels for augmented images.

### 2.5 Stages 6–7

In Stage 6, empty background pictures are selected. Numerous background images from a variety of locations and environmental conditions can improve model performance and generalization (Jocher 2020; Schneider et al. 2020). Background images do not require label files. Stage 7 splits the images in two groups: users define a fixed number of images per species for the training set, whereas the reminder will be used for validation. Consequently, training is performed with the same amount of pictures for all species, whereas there may be differences in validation set due to removals in Stage 4. Users also must define the number of background pictures for the training set, as it can differ from the number of images per species.

### 2.6 Stages 8–9

In Stage 8, training and validation of the YOLOv8 model are performed. Training is conducted first on the YOLOv8m model at a picture size of 640 pixels for 100 epochs (i.e., the number of iterations the model makes through the whole training dataset), then at 1280 pixels for 50 epochs, ensuring a faster training due to transfer learning from the first to the second step. We used default hyperparameters and online augmentation settings (Jocher, Chaurasia, and Qiu 2023). In Stage 9, the trained model is used to infer on new, unclassified images with a user‐specified detection threshold of 0.60, to reduce misdetections and misidentifications.

### 2.7 Stage 10

In Stage 10, a function calls *Exiftool* to extract the timestamp from the new pictures, and a .csv file containing the species‐level classification, date, time, full image paths, and other user‐selected details is created. Although model inference can detect multiple individuals of the same species or even more than one species in a single image, our main objective was to obtain species‐level classification of images; therefore, we attributed each image to the species identified with the highest confidence level. The database retained information regarding the second and the third species guesses, if any, based on their confidence levels.

The structure of the output database aligns with Timelapse, a software for streamline image tagging (Greenberg, Godin, and Whittington 2019). However, users can modify the database to ensure compatibility with existing datasets or other software (see Data 1 for a Timelapse‐compatible template). A commented version of the workflow with further details and the associated functions are provided as supplementary R scripts (Data 1).

### 2.8 Assisted Tagging Flow

We associated the workflow to Timelapse as it can integrate with MegaDetector (Greenberg, Godin, and Whittington 2019). Thus, even when a pre‐existing database is not immediately available, it is still possible to generate it faster than by hand through an assisted tagging process (Fennell, Beirne, and Burton 2022). First, run the MegaDetector in Stage 3 on the unprocessed images; then, incorporate the output in Timelapse and manually tag the pictures to the species levels (Figure 1, orange arrows).

### 2.9 Artificial Intelligence Disclaimer

We generated snippets of workflow code (in Workflow.R, in Data 1) and the backbone of main functions for image and labels processing (Main_functions.R, in Data 1) with ChatGPT v.3.5 (OpenAI 2024), but we reshaped AI output to achieve the desired results. Therefore, human and AI contributions are merged and undistinguishable.

### 2.10 Workflow Testing and Performance

Our workflow was tested on a set of images collected in 2021–2023 in the Apuan Alps (Central Italy) through a systematic sampling at 52 sites. Cameras were fixed on trees along trails or dirt roads at 2.5–3 m above ground to reduce the risk of theft, particularly high in the area. We first manually tagged images collected until November 2022 and then used them for training a model on 19 categories of medium to large mammals (Table 1). Sampling size was 1250 pictures per category and 3000 background images in total. Within this, the training set comprised 1000 pictures per category and 2500 background images.

**TABLE 1:** Training and validation images, and performance metrics for the best custom YOLOv8 model trained at 1280 pixels on camera trap data collected in 2021–2023 within a study on medium and large mammals in the Apuan Alps (Central Italy).

Category	Original images	Training (Augmented)	Validation (Augmented)	Precision	Recall	AP_[50–95]_
Badger ( *Meles meles* )	1250	1000 (91)	249 (22)	0.968	0.956	0.931
Cats ( *Felis silvestris* and *F. s. catus*)	1250	1000 (63)	248 (20)	0.936	0.907	0.875
Cow ( *Bos taurus* )	1038	1000 (172)	250 (46)	0.98	0.976	0.972
Dog ( *Canis lupus familiaris* )	1250	1000 (32)	250 (13)	0.97	0.904	0.913
European hare ( *Lepus europaeus* )	394	1000 (724)	149 (101)	0.994	0.998	0.89
European red squirrel ( *Sciurus vulgaris* )	737	1000 (608)	192 (113)	0.955	0.961	0.869
Fallow deer ( *Dama dama* )	1180	1000 (115)	250 (31)	0.983	0.978	0.942
Goat/sheep ( *Capra hircus* and *Ovis aries* )	1250	1000 (16)	250 (3)	0.928	0.942	0.89
Horse ( *Equus caballus* )	1012	1000 (186)	250 (67)	0.982	0.978	0.964
Humans ( *Homo sapiens* )	1250	1000 (15)	250 (4)	0.959	0.905	0.879
Mouflon ( *Ovis aries musimon* )	1250	1000 (31)	250 (7)	0.965	0.97	0.917
Other mustelids ( *Martes martes* , *M. foina* , and *Mustela putorius* )	1250	1000 (163)	249 (52)	0.976	0.953	0.913
Porcupine ( *Hystrix cristata* )	1250	1000 (103)	248 (28)	0.958	0.97	0.921
Red deer ( *Cervus elaphus* )	1250	1000 (12)	250 (2)	0.957	0.951	0.933
Red fox ( *Vulpes vulpes* )	1250	1000 (101)	248 (24)	0.932	0.888	0.879
Roe deer ( *Capreolus capreolus* )	1250	1000 (37)	250 (7)	0.978	0.93	0.932
Vehicles	1250	1000 (54)	248 (16)	0.978	0.927	0.91
Wild boar ( *Sus scrofa* )	1250	1000 (39)	250 (8)	0.939	0.935	0.914
Wolf ( *Canis lupus* )	1250	1000 (85)	249 (25)	0.941	0.931	0.897
Background		2500	500			
Overall	21,861	21,500 (2647)	5080 (589)	0.962	0.945	0.913

Model performance was assessed with precision, recall, and average precision_[50–95]_ (AP_[50–95]_) metrics calculated on the discrepancy between predictions and ground truth for the validation set precision is the ratio of true positives to the total positive predictions (true and false positives), whereas recall is a measure of true positives over the total number of actual positives (true positives and false negatives) (Padilla, Netto, and da Silva 2020). Finally, AP_[50–95]_ is the area under the precision‐recall curve across multiple thresholds of overlap with the ground truth, ranging from 0.5 to 0.95 (Padilla, Netto, and da Silva 2020).

Furthermore, a set of 15,345 unclassified test pictures was generated through a random sampling stratified by site and camera check (~330 pictures per site), and inference run on them. Model accuracy was calculated by contrasting its predictions in terms of species‐level classification of images (as defined in Stage 10) with classifications obtained by tagging all images by hand in Timelapse. Finally, the time needed to run MegaDetector, to train the YOLOv8 model, to perform inference on the new pictures, to check model predictions, and to tag images by hand was recorded.

### 2.11 Do‐It‐Yourself (DIY) Workflow

We provided a demonstrative version of the workflow as supplementary R script (Data 1), enabling users to conduct both YOLO training and inference on a small set of images collected at three distinct camera trap sites in the Apuan Alps. The DIY flow focuses on three target carnivore species: badger ( *Meles meles* ), wolf ( *Canis lupus* ), and other mustelids, where the latter were treated as a higher‐order category encompassing pine marten, stone marten, and European polecat (*Martes martes, M. foina* and *Mustela putorius* ). The sample image set is available on the first author's GitHub page (https://github.com/Lucapetroni/DIY‐YOLO‐R‐workflow‐.git).

## 3 Results

Starting from the set of 710,987 previously tagged images, a total of 21,861 pictures were extracted in Stage 2. MegaDetector identified subjects in 20,344 images (93.1%); subsequently, we performed augmentation to reach the established 1250 pictures per species. Then, MegaDetector identified subjects in 95% of the 3406 augmented images, yielding a training set of 21,500 (12.3% augmented) and a validation set of 5080 images (~250 pictures per category, 11.6% augmented) (Table 1). Background images accounted for 11.3% and 11.6% of the total and training sets, respectively.

**FIGURE 2:**
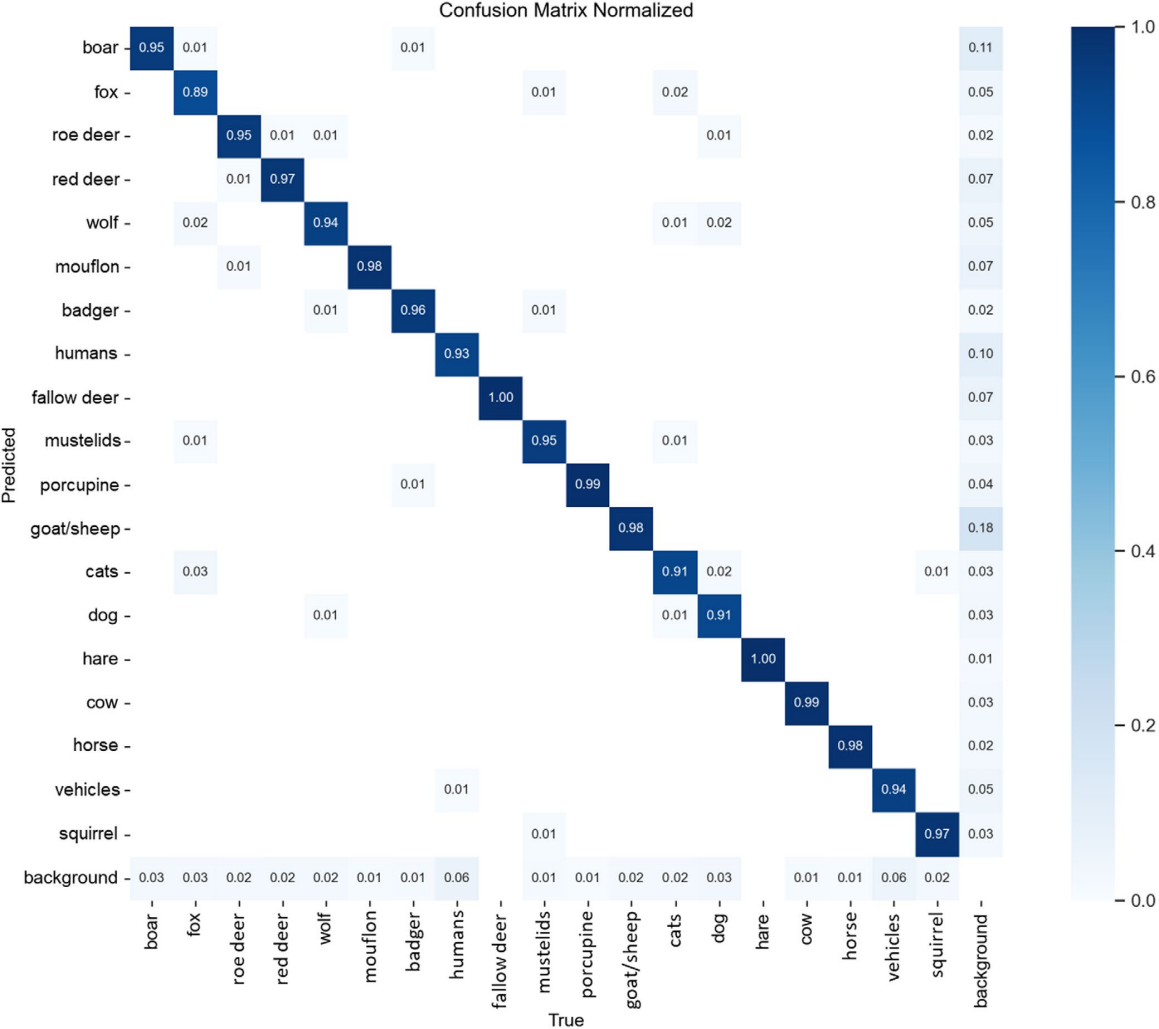
Normalized confusion matrix of correct predictions for each species obtained by the best custom YOLOv8 model for image classification at species level.

After two training steps, the model at 1280 pixels achieved an average precision of 0.962 (range: 0.928–0.994), recall of 0.945 (range: 0.888–0.998), and mean AP_[50–95]_ of 0.913 (range: 0.869–0.972) (Table 1). Red fox ( *Vulpes vulpes* ) was the only species with a recall < 0.90, whereas 7 of 19 categories had an AP_[50:95]_ of < 0.90 (Table 1). Overall, most labelling errors were represented by false positives for wild boar ( *Sus scrofa* ) and goat/sheep category, whereas the red fox was the species with the lowest proportion of correct labels at 0.89 (Figure 2). When inferring species‐level classification based on the subject tagged at the highest confidence level, model accuracy was 91.8% for the test set, and reached 97.1% for images classified with confidence > 0.90.

MegaDetector processed all the 25,267 images (i.e., the original and augmented ones) in a total of 1 h 25 min (4.95 images/s), whereas the two phases of model training required 47 h 10 min overall. Finally, inference on the test set took 1 h 34 min of unsupervised work (2.71 images/s), whereas checking of model predictions required 4 h 18 min of supervised work. Manual tagging of the same images in Timelapse required three‐fold as much time of supervised work (12:29 h).

## 4 Discussion

We developed an R‐based workflow combining YOLOv8 with offline augmentation to expedite classification of camera trap images. The workflow was tested on a dataset of images collected within a project in Central Italy, reaching an accuracy of 91.8% on new images starting from a model trained on only 1000 already tagged images per species, considered the minimal requisite to obtain reliable results (Schneider et al. 2020).

Our results were consistent with other recent studies indicating the potential of YOLO models in species‐level classification of camera trap images (Rigoudy et al. 2023; Tan et al. 2022). Notably, we lacked 1000 tagged images for two target species (European hare and European red squirrel) and > 10% of the training and validation images were created through offline augmentation. Finally, the model processed the test set in less than 2 h, and, most importantly, without supervision, whereas manual checking of model predictions required ~34% of the time needed to tag images by hand.

Thanks to offline augmentation, the workflow can streamline image classification even in the early stages of a project when fewer images are available, and model performance can be improved with subsequent re‐training. Also, the light and fast architecture enables training on resource‐limited machines, although it is still advisable to perform training on deep learning compatible GPU to hasten the process. Once a custom model is obtained, inference on new data can be performed on a laptop directly in the field, potentially reducing image storage requirements (i.e., removal of empty pictures) and costs in terms of memory cards.

The custom model obtained an accuracy of 97.1% for test images classified with > 90% confidence. Establishing confidence thresholds for accepting model predictions can dramatically hasten image processing. Böhner et al. (2023) reported an accuracy of 99% for images classified with a confidence > 90% and estimated to have saved 70 h of human work by accepting those labels. Such promising results probably do not represent a standalone solution to automate image classification and analysis (Lonsinger et al. 2024), but could provide the basis for integration of deep learning with statistical approaches accounting for false positives (Chambert, Miller, and Nichols 2015) or confidence values (Rhinehart, Turek, and Kitzes 2022), although human review of images identified below the confidence threshold will still be needed.

We are unaware of other classifiers trained on images of Italian wildlife. Recently, pretrained models for European mammals emerged, but their application usually requires Python skills (Carl et al. 2020; Choiński et al. 2021), and software is limited to inference tasks using existing models (Rigoudy et al. 2023). Our pretrained models (Data 1) may represent a starting point for inference in other studies carried out in similar ecological contexts or on comparable arrays of species. Our camera traps were placed 2.5–3 m above the ground and we expect lower performance in contexts where cameras are set at different heights. Custom training may be required, but could still benefit from transfer learning from our pretrained model.

Although our workflow is currently limited to pictures rather than videos, it could be easily adapted to train models on video frames (Tan et al. 2022). Additionally, proficient MacOS or Linux users may implement it by adjusting installation procedures in Stage 1 and model related functions in Stages 3, 8, and 9. However, the Timelapse software is currently exclusive to Windows. Hence, users working in different systems may need to customize Stage 10 to generate data frames compatible with other image handling software and verify model predictions.

In summary, the proposed workflow may represent a comprehensive and versatile tool to facilitate deep learning implementation for ecologists familiar with the R language, and expedite image processing in long term ecological studies.

## Author Contributions

**L. Petroni:** conceptualization (equal), data curation (equal), formal analysis (lead), methodology (lead), writing – original draft (equal), writing – review and editing (equal). **L. Natucci:** data curation (equal), formal analysis (supporting), methodology (supporting), writing – original draft (supporting), writing – review and editing (supporting). **A. Massolo:** conceptualization (equal), methodology (supporting), supervision (lead), writing – original draft (equal), writing – review and editing (equal).

## Conflicts of Interest

The authors declare no conflicts of interest.

## Supporting information


Data S1.

## Data Availability

All scripts, an example database for camera trap metadata useful for the Do‐It‐Yourself (DIY) version of the workflow, an example of Timelapse template compatible with the workflow, and pretrained deep learning models are in Supporting Information. Sample images to implement the DIY version of the workflow are available at the first author's GitHub page (https://github.com/Lucapetroni/DIY‐YOLO‐R‐workflow‐.git).
